# Small deviations for admixture additive & multiplicative processes

**DOI:** 10.1186/s13660-018-1798-4

**Published:** 2018-08-08

**Authors:** Mingjie Liang, Bingyao Wu

**Affiliations:** College of Mathematics and Informatics, Fujian Normal University, Fuzhou, P.R. China

**Keywords:** 60F15, 60G60, Small deviation, Admixture additive process, Admixture multiplicative process, Limit theorem

## Abstract

Define the admixture additive processes

$$\mathbb{X}^{a_{1}, a_{2}, a_{3}, a_{4}}_{\gamma,H,\alpha}(\mathrm{t})\triangleq a_{1}B(t_{1})+a_{2}W_{\gamma}(t_{2})+a_{3}B_{H}(t_{3})+a_{4}S_{\alpha}(t_{4}) \in\mathbb{R}, $$

and the admixture multiplicative processes

$$\mathbb{Y}_{\gamma,H,\alpha}(\mathrm{t})\triangleq B(t_{1})\cdot W_{\gamma}(t_{2})\cdot B_{H}(t_{3})\cdot S_{\alpha}(t_{4})\in\mathbb{R}, $$

where $\mathrm{t}=(t_{1},t_{2},t_{3},t_{4})\in\mathbb{R}_{+}^{\mathrm{4}},a_{1},a_{2},a_{3},a_{4}$ are finite constants, $B(t_{1})$ is the standard Brownian motion, $W_{\gamma}(t_{2})$ is the fractional integrated Brownian motion with index parameter $\gamma>-1/2$, $B_{H}(t_{3})$ is the fractional Brownian motion with Hurst parameter $H\in(0,1)$, $S_{\alpha}(t_{4})$ is the stable process with index $\alpha\in(0,2]$, and they are independent of each other. The small deviation for $\mathbb{X}^{a_{1}, a_{2}, a_{3}, a_{4}}_{\gamma,H,\alpha}(\mathrm{t})$ and the lower bound of small deviation for $\mathbb{Y}_{\gamma,H,\alpha}(\mathrm{t})$ are obtained. As an application, limit inf type LIL is given for $\mathbb{X}^{a_{1}, a_{2}, a_{3}, a_{4}}_{\gamma,H,\alpha}(\mathrm{t})$.

## Introduction and main results

Let $B= \{B(t_{1})\in\mathbb{R},t_{1}\in\mathbb{R}_{+} \}$ be a standard Brownian motion, $W_{\gamma}= \{W_{\gamma}(t_{2})\in \mathbb{R}, t_{2}\in\mathbb{R}_{+} \}$ be a fractional integrated Brownian motion with index parameter $\gamma>-1/2$, $B_{H}= \{B_{H}(t_{3})\in \mathbb{R},t_{3}\in\mathbb{R}_{+} \}$ be a fractional Brownian motion with Hurst parameter $H\in(0,1)$, $S_{\alpha}= \{S_{\alpha}(t_{4})\in\mathbb{R},t_{4}\in\mathbb{R}_{+} \}$ be a stable process with index $\alpha\in(0,2]$, and they are independent of each other. Define the admixture additive processes

$$\begin{aligned} \mathbb{X}^{a_{1}, a_{2}, a_{3}, a_{4}}_{\gamma,H,\alpha}(\mathrm{t})\triangleq a_{1}B(t_{1})+a_{2}W_{\gamma}(t_{2})+a_{3}B_{H}(t_{3})+a_{4}S_{\alpha}(t_{4}) \in\mathbb{R}, \end{aligned}$$

and the admixture multiplicative processes

$$\begin{aligned} \mathbb{Y}_{\gamma,H,\alpha}(\mathrm{t})\triangleq B(t_{1})\cdot W_{\gamma}(t_{2})\cdot B_{H}(t_{3})\cdot S_{\alpha}(t_{4})\in\mathbb{R}, \end{aligned}$$

where $\mathrm{t}=(t_{1},t_{2},t_{3},t_{4})\in\mathbb{R}_{+}^{\mathrm{4}}$ and $a_{1},a_{2},a_{3},a_{4}$ are finite constants.

### Remark 1.1

In particular, $W_{0}(t)=B_{1/2}(t)=S_{2}(t)$ is the standard Brownian motion. Quite obviously, the process $\mathbb{X}^{1, 1, 1, 1}_{0,1/2,2}(\mathrm{t})$ is the additive Brownian motions. For more details on the additive Brownian motions, the reader can refer to the monographs [1] and [2].

The object of study in this paper will be the small deviations for $\mathbb{X}^{a_{1}, a_{2}, a_{3}, a_{4}}_{\gamma,H,\alpha}(\mathrm{t})$ and $\mathbb{Y}_{\gamma,H,\alpha}(\mathrm{t})$ which are formally defined as follows:

$$\begin{aligned} \log\mathbb{P} \Bigl(\sup_{\mathrm{t}\in[0,1]^{4}} \vert \cdot \vert \leq \varepsilon \Bigr)=-\phi(\varepsilon)\quad \mbox{as }\varepsilon \rightarrow 0. \end{aligned}$$

Our standard reference is the monograph [3]. There are a number of papers concerned with the small deviations for various stochastic processes. For details, we refer to the monographs [3, 4]. If properly defined, the small deviation in some literatures is called the small ball probability (see, e.g., [5, 6]). There are various motivations for the study of some additive processes, and it has been actively investigated recently from different points of view, see Khoshnevisan, Xiao, and Zhong [2, 7] for a detailed discussion and the bibliography for further works in this area. First of all, additive processes play an important role in the study of other more interesting multiparameter processes since they locally resemble multiparameter processes, such as Brownian sheet, fractional Brownian sheet, and stable sheet, and also because they are more amenable to analysis. For example, locally and with time suitably rescaled, the Brownian sheet closely resembles an additive Brownian motion (see, e.g., [8, 9]). They also arise in the theory of intersection and self-intersection local times of Brownian processes (see, e.g.,[10, 11]).

Now, we briefly give the small deviation estimates for the admixture additive processes $\mathbb{X}^{a_{1}, a_{2}, a_{3}, a_{4}}_{\gamma,H,\alpha}(\mathrm{t})$ and the admixture multiplicative processes $\mathbb{Y}_{H,\gamma,\alpha}(\mathrm{t})$. The main results are the following.

### Theorem 1.2

1.1 $$\begin{aligned} \lim_{\varepsilon\rightarrow 0^{+}}\varepsilon^{\beta}\log \mathbb{P} \Bigl(\sup_{\mathrm{t}\in [0,1]^{4}} \bigl\vert \mathbb{X}^{a_{1}, a_{2}, a_{3}, a_{4}}_{\gamma,H,\alpha}( \mathrm{t}) \bigr\vert \leq \varepsilon \Bigr)=-\vartheta (\gamma, H, \alpha, a_{1}, a_{2}, a_{3}, a_{4} ) \end{aligned}$$

*and*

1.2 $$\begin{aligned} \liminf_{\varepsilon\rightarrow 0^{+}}\varepsilon^{\beta}\log \mathbb{P} \Bigl(\sup_{\mathrm{t}\in[0,1]^{4}} \bigl\vert \mathbb{Y}_{\gamma,H,\alpha}( \mathrm{t}) \bigr\vert \leq \varepsilon \Bigr)\geq -\nu (\gamma, H, \alpha ), \end{aligned}$$

*where* $\beta=\max \{2,\frac{2}{2\gamma+1},\frac{1}{H} \}$, $\vartheta (\gamma, H, \alpha, a_{1}, a_{2}, a_{3}, a_{4} )= ( (\operatorname{sgn}(2-\beta)+1 ) (\frac {(a_{1}\pi)^{2}}{8} )^{\frac{1}{1+\beta}}+ (\operatorname{sgn}(2/ (2\gamma+1)- \beta)+1 ) (a_{2}^{2/(2\gamma+1)}k_{\gamma})^{\frac{1}{1+\beta}} + (\operatorname{sgn}(1/H-\beta)+1 ) (|a_{3}|^{1/H}C_{H} )^{\frac{1}{1+\beta}} + (\operatorname{sgn}(\alpha-\beta)+1 ) (|a_{4}|^{\alpha}A_{\alpha})^{\frac{1}{1+\beta}} )^{1+\beta},\nu (\gamma, H, \alpha )= (\operatorname{sgn}(2-\beta)+1 )\frac{\pi^{2}}{8} + (\operatorname{sgn}(2/(2\gamma+1)-\beta)+1 )k_{\gamma}+ (\operatorname{sgn}(1/H-\beta)+1 )C_{H} + (\operatorname{sgn}(\alpha-\beta)+1 )A_{\alpha}$. *In particular*, $\vartheta (0,1/2,2,1,1,1,1 )=8\pi^{2}, \nu (0, 1/2, 2 )=-\frac{\pi^{2}}{2}$.

### Remark 1.3

Unfortunately, we just obtain the lower bound of the small deviation for the admixture multiplicative processes. Certainly, it is easy to get the following upper and lower bounds of the small deviation in the special case $\gamma=0,H=1/2,\alpha=2$,

$$\begin{aligned} \limsup_{\varepsilon\rightarrow 0^{+}}\varepsilon^{1/2}\log\mathbb{P} \Bigl( \sup_{\mathrm{t}\in[0,1]^{4}} \bigl\vert \mathbb{Y}_{0,1/2,2}(\mathrm{t}) \bigr\vert \leq \varepsilon \Bigr)\leq -\frac{\pi^{2}}{8} \end{aligned}$$

and

$$\begin{aligned} \liminf_{\varepsilon\rightarrow 0^{+}}\varepsilon^{1/2}\log\mathbb{P} \Bigl( \sup_{\mathrm{t}\in[0,1]^{4}} \bigl\vert \mathbb{Y}_{0,1/2,2}(\mathrm{t}) \bigr\vert \leq \varepsilon \Bigr)\geq -\frac{\pi^{2}}{2}. \end{aligned}$$

We observe that the admixture multiplicative process $\mathbb{Y}_{0,1/2,2}(\mathrm{t})$ has the same covariance function with $(4,1)$-Brownian sheet $\{X(\mathrm{t})\in\mathbb{R},\mathrm{t}\in\mathbb {R}_{+}^{\mathrm{4}} \}$. Naturally, are there some similar properties between them? In fact, the small deviation for $(4,1)$-Brownian sheet is much more difficult to discuss. The fact that in [4] Wenbo V. Li and Qi-Man Shao only obtained the upper and lower bounds of the small ball probability for $(4,1)$-Brownian sheet,

$$\begin{aligned} -K_{2}\varepsilon^{-2}\log^{7}(1/\varepsilon) \leq \log\mathbb {P} \Bigl(\sup_{\mathrm{t}\in[0,1]^{4}} \bigl\vert X( \mathrm{t}) \bigr\vert \leq \varepsilon \Bigr)\leq -K_{1} \varepsilon^{-2}\log^{6}(1/\varepsilon), \end{aligned}$$

where $0< K_{1},K_{2}<\infty$.

By Lemma 2.1 in [5] and (1.1) in Theorem 1.2, we deduce the following corollary.

### Corollary 1.4

$$\begin{aligned} \lim_{\lambda\rightarrow\infty}\lambda^{\beta/(1+\beta)}\log \mathbb{E} \exp \Bigl(- \lambda\sup_{\mathrm{t}\in[0,1]^{4}} \bigl\vert \mathbb{X}^{a_{1}, a_{2}, a_{3}, a_{4}}_{\gamma,H,\alpha}( \mathrm{t}) \bigr\vert \Bigr)=-(1+\beta)\beta ^{-\beta/(1+\beta)} \vartheta^{1/(1+\beta)}, \end{aligned}$$

*where* $\vartheta (\gamma, H, \alpha, a_{1}, a_{2}, a_{3}, a_{4} )$ *is denoted as* *ϑ* *for convenience*.

### Remark 1.5

The result of above Corollary 1.4 seems to be a special case of the so-called de Bruijin’s exponential Tauberian theorem by Bingham et al. For details, it establishes the relationship between the asymptotic behavior of Laplace transform and the small deviation for the admixture additive processes $\mathbb{X}^{a_{1}, a_{2}, a_{3}, a_{4}}_{\gamma,H,\alpha}(\mathrm{t})$.

As an application of the general results for Theorem 1.2, the below theorem shows the Chung-type law of the iterated logarithm (LIL) for the admixture additive processes $\mathbb{X}^{a_{1}, a_{2}, a_{3}, a_{4}}_{\gamma,H,\alpha}(\mathrm{t})$.

### Theorem 1.6

1.3 $$\begin{aligned} \liminf_{T\rightarrow\infty} \bigl(T^{-1}\log\log T \bigr)^{1/\beta}\sup_{\mathrm{t}\in[0,T]^{4}} \bigl\vert \mathbb{X}^{a_{1}, a_{2}, a_{3}, a_{4}}_{\gamma,H,\alpha}(\mathrm{t}) \bigr\vert = \vartheta^{1/\beta } (\gamma, H, \alpha, a_{1}, a_{2}, a_{3}, a_{4} ). \end{aligned}$$

### Remark 1.7

Since we have the lower bound of small deviation for $\mathbb{Y}_{\gamma,H,\alpha}(\mathrm{t})$ only, the limit inf type LIL for $\mathbb{Y}_{\gamma,H,\alpha}(\mathrm{t})$ cannot be obtained. In fact, using an argument similar to that given in the proof of (1.3), we can obtain

1.4 $$\begin{aligned} \liminf_{T\rightarrow\infty} \bigl(T^{-1}\log\log T \bigr)^{1/\beta}\sup_{\mathrm{t}\in[0,1]^{4}} \bigl\vert \mathbb{Y}_{\gamma,H,\alpha}(\mathrm{t}) \bigr\vert \geq \nu^{1/\beta} ( \gamma, H, \alpha ). \end{aligned}$$

Furthermore, we also can consider a generalization of the admixture additive processes

$$\begin{aligned} \mathbb{X}^{\mathbf{a}_{1}, \mathbf{a}_{2}, \mathbf{a}_{3}, \mathbf{a}_{4}}_{\boldsymbol{\gamma},\mathbf{H},\boldsymbol{\alpha }}(\mathrm{t},\mathrm{N}_{1}, \mathrm{N}_{2},\mathrm{N}_{3},\mathrm{N}_{4}) \triangleq{}& \sum_{i=1}^{\mathrm{N}_{1}}a_{1i}B^{i}(t_{1i})+ \sum_{j=1}^{\mathrm{N}_{2}}a_{2j}W^{j}_{\gamma_{j}}(t_{2j}) \\ &{} + \sum_{k=1}^{\mathrm{N}_{3}}a_{3k}B^{k}_{H_{k}}(t_{3k})+ \sum_{l=1}^{\mathrm{N}_{4}}a_{4l}S^{l}_{\alpha_{l}}(t_{4l}), \end{aligned}$$

and the admixture multiplicative processes

$$\begin{aligned} \mathbb{Y}_{\boldsymbol{\gamma},\mathbf{H},\boldsymbol{\alpha}}(\mathrm {t},\mathrm{N}_{1}, \mathrm{N}_{2},\mathrm{N}_{3},\mathrm{N}_{4}) \triangleq \prod_{i=1}^{\mathrm{N}_{1}}B^{i}(t_{1i}) \cdot \prod_{j=1}^{\mathrm{N}_{2}}W^{j}_{\gamma_{j}}(t_{2j}) \cdot \prod_{k=1}^{\mathrm{N}_{3}}a_{3k}B^{k}_{H_{k}}(t_{3k}) \cdot \prod_{l=1}^{\mathrm{N}_{4}}a_{4l}S^{l}_{\alpha_{l}}(t_{4l}). \end{aligned}$$

Obviously, there are some results in common with those given in Theorems 1.2, 1.6, and we omit the details.

The remainder of the paper is arranged as follows. We present some preliminaries and the basic lemmas for establishing the small deviations of the admixture additive processes $\mathbb{X}^{a_{1}, a_{2}, a_{3}, a_{4}}_{\gamma,H,\alpha}(\mathrm{t})$ and the admixture multiplicative processes $\mathbb{Y}_{H,\gamma,\alpha}(\mathrm{t})$ in Sect. 2. The proofs of the main results are given in Sect. 3.

## Preliminaries

Firstly, we briefly recall the processes which are the compositions of constructing the admixture additive processes $\mathbb{X}^{a_{1}, a_{2}, a_{3}, a_{4}}_{\gamma,H,\alpha}(\mathrm{t})$ and the admixture multiplicative processes $\mathbb{Y}_{\gamma,H,\alpha}(\mathrm{t})$.

The standard Brownian motion $B= \{B(t)\in\mathbb{R},t\in \mathbb{R}_{+} \}$ with $B(0)=0$ specifies

$$B(t)-B(s)\thicksim\mathrm{N}(0,t-s), \quad \forall 0\leq s< t. $$

The fractional integrated Brownian motion $W_{\gamma}= \{W_{\gamma}(t)\in\mathbb{R},t\in\mathbb{R}_{+} \}$ is defined by

$$W_{\gamma}=\frac{1}{\Gamma(\gamma+1)} \int_{0}^{t}(t-s)^{\gamma}\,\mathrm {d}B(s) \quad( \gamma>-1/2), $$

where $\Gamma(z)=\int_{0}^{\infty}x^{z-1}e^{-x}\,\mathrm{d}x$ is the gamma function and $B(s)$ is a real-valued standard Brownian motion.

The fractional Brownian motion $B_{H}= \{B_{H}(t)\in \mathbb{R},t\in\mathbb{R}_{+} \}$ with $B_{H}(0)=0$ is a Gaussian process which has mean zero and the following covariance function:

$$\mathbb{E} \bigl(B_{H}(t)B_{H}(s) \bigr)=\frac{1}{2} \bigl( \vert t \vert ^{2H}+ \vert s \vert ^{2H}- \vert t-s \vert ^{2H} \bigr)\quad (0< H< 1). $$

The stable process $S_{\alpha}= \{S_{\alpha}(t)\in\mathbb{R},t\in\mathbb{R}_{+} \}$ with index $\alpha\in(0,2]$ has the characteristic function $\mathbb{E} (e^{\mathrm{i}(u,S_{\alpha}(t))} )=e^{-t\psi(u)}$ whose exponent $\psi(u)$ has the following form:

$$\psi(u)=\mathrm{i}(a,u)+\lambda \vert u \vert ^{\alpha}\int_{\mathfrak {S}_{\mathbf{d}}}\widetilde{W}_{\alpha}(u,\theta)\mu(\mathrm{d} \theta), $$

where $\widetilde{W}_{\alpha}(u,\theta)$ satisfies

$$\begin{aligned} \widetilde{W}_{\alpha}(u,\theta)= \textstyle\begin{cases} [1- \mathrm{i} \operatorname{sgn}(u,\theta)\tan\frac{\pi\alpha }{2} ] \vert (\frac{u}{ \vert u \vert },\theta ) \vert ^{\alpha},& \alpha \neq1, \\ \vert (\frac{u}{ \vert u \vert },\theta ) \vert + \frac{2\mathrm {i}}{\pi}(u,\theta)\log \vert (u,\theta) \vert ,& \alpha=1. \end{cases}\displaystyle \end{aligned}$$

Specifically, if $\psi(u)=\lambda|u|^{\alpha}$, the process $S_{\alpha}(t)$ is called the symmetric stable process. For more details on the above processes, the reader can refer to the monographs [12, 13].

In order to establish the estimates of the small deviations conveniently, we present some lemmas at first. In fact, Lemmas 2.1–2.4 show the relation between the small deviation estimates of sup-norm and the range for the Brownian motion, the fractional integrated Brownian motion, the fractional Brownian motion, and the stable process, respectively.

### Lemma 2.1

*Let* $\{B(t)\in\mathbb{R},t\in\mathbb{R}_{+} \}$ *be a standard Brownian motion*, *then for any given finite constant* $a_{1}\in\mathbb{R}$,

2.1 $$\begin{aligned} \lim_{\varepsilon\rightarrow 0}\varepsilon^{2}\log \mathbb{P} \Bigl(\sup_{t\in[0,1]} \bigl\vert a_{1}B(t) \bigr\vert \leq \varepsilon \Bigr)=-\frac{(a_{1}\pi)^{2}}{8}. \end{aligned}$$

*Then*, *for the range* $R_{1}=\sup_{s,t\in[0,1]} \vert B(t)-B(s) \vert $, *we have*

2.2 $$\begin{aligned} \lim_{\varepsilon\rightarrow 0}\varepsilon^{2}\log \mathbb{P} (R_{1}\leq \varepsilon )=-\frac{ (2\pi)^{2}}{8}. \end{aligned}$$

### Lemma 2.2

*Let* $\{W_{\gamma}(t)\in\mathbb{R},t\in\mathbb{R}_{+} \}$ *be a fractional integrated Brownian motion*, *then for any given finite constant* $a_{2}\in\mathbb{R}$,

2.3 $$\begin{aligned} \lim_{\varepsilon\rightarrow 0}\varepsilon^{2/(2\gamma+1)}\log \mathbb{P} \Bigl(\sup_{t\in [0,1]} \bigl\vert a_{2}W_{\gamma}(t) \bigr\vert \leq \varepsilon \Bigr)=-a_{2}^{2/(2\gamma+1)}k_{\gamma}, \end{aligned}$$

*where* $k_{\gamma}\in(0,\infty)$ *is given by*

$$\begin{aligned} k_{\gamma}=-\inf_{\varepsilon>0}\varepsilon^{2/(2\gamma+1)}\log \mathbb{P} \Bigl(\sup_{0\leq t\leq 1} \bigl\vert W_{\gamma}(t) \bigr\vert \leq \varepsilon \Bigr). \end{aligned}$$

*Then*, *for the range* $R_{2}=\sup_{s,t\in[0,1]} \vert W_{\gamma}(t)-W_{\gamma}(s) \vert $, *we have*

2.4 $$\begin{aligned} \lim_{\varepsilon\rightarrow 0}\varepsilon^{2/(2\gamma+1)}\log \mathbb{P} (R_{2}\leq \varepsilon )=-2^{2/(2\gamma+1)}k_{\gamma}. \end{aligned}$$

### Lemma 2.3

*Let* $\{B_{H}(t)\in \mathbb{R},t\in\mathbb{R}_{+} \}$ *be a fractional Brownian motion*, *then for any given finite constant* $a_{3}\in\mathbb{R}$,

2.5 $$\begin{aligned} \lim_{\varepsilon\rightarrow 0}\varepsilon^{1/H}\log \mathbb{P} \Bigl(\sup_{t\in[0,1]} \bigl\vert a_{3}B_{H}(t) \bigr\vert \leq \varepsilon \Bigr)=- \vert a_{3} \vert ^{1/H}C_{H}, \end{aligned}$$

*where* $C_{H}\in(0,\infty)$ *is given by*

$$\begin{aligned} C_{H}=k_{H-1/2}\cdot \bigl(\Gamma(H+1/2) \bigr)^{1/H} \cdot \biggl(\frac{1}{2H}+ \int_{-\infty}^{0} \bigl((1-s)^{H-1/2}-(-s)^{H-1/2} \bigr)^{2}\,\mathrm {d}s \biggr)^{-1/(2H)}. \end{aligned}$$

*Then*, *for the range* $R_{3}=\sup_{s,t\in[0,1]} \vert B_{H}(t)-B_{H}(s) \vert $, *we have*

2.6 $$\begin{aligned} \lim_{\varepsilon\rightarrow 0}\varepsilon^{1/H}\log \mathbb{P} (R_{3}\leq \varepsilon )=-2^{1/H}C_{H}. \end{aligned}$$

### Lemma 2.4

*Let* $\{S_{\alpha}(t)\in\mathbb{R},t\in \mathbb{R}_{+} \}$ *be a stable process*, *then for any given finite constant* $a_{4}\in\mathbb{R}$,

2.7 $$\begin{aligned} \lim_{\varepsilon\rightarrow 0}\varepsilon^{\alpha}\log \mathbb{P} \Bigl(\sup_{t\in[0,1]} \bigl\vert a_{4}S_{\alpha}(t) \bigr\vert \leq \varepsilon \Bigr)=- \vert a_{4} \vert ^{\alpha}A_{\alpha}, \end{aligned}$$

*where* $A_{\alpha}>0$ *is the principle Dirichlet eigenvalue for the fractional Laplacian operator associated with* $S_{\alpha}(t)$ *in the interval* $[-1,1]$. *Then*, *for the range* $R_{4}=\sup_{s,t\in[0,1]} \vert S_{\alpha}(t)-S_{\alpha}(s) \vert $, *we have*

2.8 $$\begin{aligned} \lim_{\varepsilon\rightarrow 0}\varepsilon^{\alpha}\log \mathbb{P} (R_{4}\leq \varepsilon )=-2^{\alpha}A_{\alpha}. \end{aligned}$$

### Remark 2.5

It seems to be little known about the explicit value of $A_{\alpha}$ in Lemma 2.4, $0<\alpha<2$, although sometimes this constant appears in some other problems. The best known bounds of $A_{\alpha}$ for a symmetric stable process whose characteristic function exponent $\psi(u)=K|u|^{\alpha}$ are

$$\begin{aligned} \Gamma(\alpha+1)\leq A_{\alpha} \leq \Gamma \biggl( \frac{\alpha}{2} \biggr)\Gamma \biggl(\alpha+\frac {3}{2} \biggr) \Big/\Gamma \biggl(\frac{\alpha+3}{2} \biggr) \quad(0< \alpha< 2), \end{aligned}$$

and it is a challenge to find more explicit expression for $A_{\alpha}$ than the well-known variation one.

### Remark 2.6

Lemmas 2.1–2.4 are proved easily by the well-known results in [3]. In the case of the Brownian motions, it is well known that $k_{0}=C_{1/2}=A_{2}=\frac{ \pi^{2}}{8}$.

## Proof of the main results

We give the proof of the main results in this section. An inspection of our arguments reveals that the special structures of admixture additive and multiplicative processes play a very important role in the following derivations. A key ingredient of our approach is with reference to Chen and Li [3].

### Proof of (1.1) in Theorem 1.2

We follow similar approaches and steps in the proof of Theorem 5.2 as those in [3] and Lemma 2 in [5]. For any given finite constants $a_{1},a_{2},a_{3},a_{4}\in\mathbb{R}$, using the triangle inequality, we have

$$\begin{aligned} &\sup_{\mathrm{t}\in[0,1]^{4}} \bigl\vert \mathbb{X}^{a_{1}, a_{2}, a_{3}, a_{4}}_{\gamma,H,\alpha}( \mathrm{t}) \bigr\vert \\ &\quad \leq \sup_{(t_{1},t_{2},t_{3},t_{4})\in[0,1]^{4}} \bigl( \bigl\vert a_{1}B(t_{1}) \bigr\vert + \bigl\vert a_{2}W_{\gamma}(t_{2}) \bigr\vert + \bigl\vert a_{3}B_{H}(t_{3}) \bigr\vert + \bigl\vert a_{4}S_{\alpha}(t_{4}) \bigr\vert \bigr) \\ &\quad = \sup_{t_{1}\in[0,1]} \bigl\vert a_{1}B(t_{1}) \bigr\vert + \sup_{t_{2}\in[0,1]} \bigl\vert a_{2}W_{\gamma}(t_{2}) \bigr\vert + \sup_{t_{3}\in[0,1]} \bigl\vert a_{3}B_{H}(t_{3}) \bigr\vert + \sup_{t_{4}\in[0,1]} \bigl\vert a_{4}S_{\alpha}(t_{4}) \bigr\vert . \end{aligned}$$

Thus

3.1 $$\begin{aligned} &\mathbb{P} \Bigl(\sup_{\mathrm{t}\in[0,1]^{4}} \bigl\vert \mathbb{X}^{a_{1}, a_{2}, a_{3}, a_{4}}_{\gamma,H,\alpha}(\mathrm{t}) \bigr\vert \leq \varepsilon \Bigr) \\ &\quad \geq \mathbb{P} \Bigl(\sup_{t_{1}\in[0,1]} \bigl\vert a_{1}B(t_{1}) \bigr\vert + \sup_{t_{2}\in[0,1]} \bigl\vert a_{2}W_{\gamma}(t_{2}) \bigr\vert \\ &\qquad{} +\sup_{t_{3}\in[0,1]} \bigl\vert a_{3}B_{H}(t_{3}) \bigr\vert + \sup_{t_{4}\in[0,1]} \bigl\vert a_{4}S_{\alpha}(t_{4}) \bigr\vert \leq \varepsilon \Bigr). \end{aligned}$$

Without loss of generality, we argue the following condition: $\gamma+1/2< H\leq 1/2\leq 1/\alpha$, in this case $\beta=2/(2\gamma+1)$.

On the one hand, using (3.1) for fixed $\delta>0$ small enough, we have

3.2 $$\begin{aligned} &\mathbb{P} \Bigl(\sup_{\mathrm{t}\in[0,1]^{4}} \bigl\vert \mathbb{X}^{a_{1}, a_{2}, a_{3}, a_{4}}_{\gamma,H,\alpha}(\mathrm{t}) \bigr\vert \leq \varepsilon \Bigr) \\ &\quad \geq \mathbb{P} \Bigl( \Bigl\{ \sup_{t_{2}\in[0,1]} \bigl\vert a_{2}W_{\gamma}(t_{2}) \bigr\vert \leq a_{2}^{2/(2\gamma+3)}k_{\gamma}^{(2\gamma+1)/(2\gamma+3)}\varepsilon /C(a_{2},k_{\gamma},\delta) \Bigr\} \\ &\qquad{} \cap \Bigl\{ \sup_{t_{1}\in[0,1]} \bigl\vert a_{1}B(t_{1}) \bigr\vert \leq \delta\varepsilon /C(a_{2},k_{\gamma}, \delta) \Bigr\} \\ &\qquad{} \cap \Bigl\{ \sup_{t_{3}\in[0,1]} \bigl\vert a_{3}B_{H}(t_{3}) \bigr\vert \leq \delta \varepsilon /C(a_{2},k_{\gamma}, \delta) \Bigr\} \\ &\qquad{} \cap \Bigl\{ \sup_{t_{4}\in[0,1]} \bigl\vert a_{4}S_{\alpha}(t_{4}) \bigr\vert \leq \delta \varepsilon /C(a_{2},k_{\gamma},\delta ) \Bigr\} \Bigr) \\ &\quad =\mathbb{P} \Bigl(\sup_{t_{2}\in[0,1]} \bigl\vert a_{2}W_{\gamma}(t_{2}) \bigr\vert \leq a_{2}^{2/(2\gamma+3)}k_{\gamma}^{(2\gamma+1)/(2\gamma+3)}\varepsilon /C(a_{2},k_{\gamma},\delta) \Bigr) \\ &\qquad{}\times\mathbb{P} \Bigl(\sup_{t_{1}\in[0,1]} \bigl\vert a_{1}B(t_{1}) \bigr\vert \leq \delta\varepsilon /C(a_{2},k_{\gamma},\delta) \Bigr) \\ &\qquad{}\times\mathbb{P} \Bigl(\sup_{t_{3}\in[0,1]} \bigl\vert a_{3}B_{H}(t_{3}) \bigr\vert \leq \delta \varepsilon /C(a_{2},k_{\gamma},\delta) \Bigr) \\ &\qquad{}\times\mathbb{P} \Bigl(\sup_{t_{4}\in[0,1]} \bigl\vert a_{4}S_{\alpha}(t_{4}) \bigr\vert \leq \delta \varepsilon /C(a_{2},k_{\gamma},\delta ) \Bigr), \end{aligned}$$

where $C(a_{2},k_{\gamma},\delta)=a_{2}^{2/(2\gamma+3)}k_{\gamma}^{(2\gamma +1)/(2\gamma+3)} +4\delta$.

Then, combining (2.1), (2.3), (2.5), and (2.7) with (3.2), we get

3.3 $$\begin{aligned} &\liminf_{\varepsilon\rightarrow 0^{+}}\varepsilon^{\beta}\log \mathbb{P} \Bigl(\sup_{\mathrm{t}\in [0,1]^{4}} \bigl\vert \mathbb{X}^{a_{1}, a_{2}, a_{3}, a_{4}}_{\gamma,H,\alpha}( \mathrm{t}) \bigr\vert \leq \varepsilon \Bigr) \\ &\quad \geq -a_{2}^{2/(2\gamma+3)}k_{\gamma}^{(2\gamma+1)/(2\gamma+3)} \bigl(a_{2}^{2/(2\gamma+3)}k_{\gamma}^{(2\gamma+1)/(2\gamma+3)} +4\delta \bigr)^{2/(2\gamma+1)}. \end{aligned}$$

Taking $\delta\rightarrow0$ in (3.3), we obtain that

$$\begin{aligned} \liminf_{\varepsilon\rightarrow 0^{+}}\varepsilon^{\beta}\log \mathbb{P} \Bigl(\sup_{\mathrm{t}\in [0,1]^{4}} \bigl\vert \mathbb{X}^{a_{1}, a_{2}, a_{3}, a_{4}}_{\gamma,H,\alpha}( \mathrm{t}) \bigr\vert \leq \varepsilon \Bigr)\geq -a_{2}^{2/(2\gamma+1)}k_{\gamma}. \end{aligned}$$

On the other hand, we observe that

$$\begin{aligned} \sum_{i=1}^{4} \vert a_{i} \vert R_{i}={}&\sup_{s,t\in[0,1]} \bigl\vert a_{1}B(t)-a_{1}B(s) \bigr\vert +\sup _{s,t\in[0,1]} \bigl\vert a_{2}W_{\gamma}(t)-a_{2}W_{\gamma}(s) \bigr\vert \\ &{}+\sup_{s,t\in[0,1]} \bigl\vert a_{3}B_{H}(t)-a_{3}B_{H}(s) \bigr\vert + \sup_{s,t\in[0,1]} \bigl\vert a_{4}S_{\alpha}(t)-a_{4}S_{\alpha}(s) \bigr\vert \\ ={}& \Bigl(\sup_{t\in[0,1]}a_{1}B(t)-\inf _{t\in[0,1]}a_{1}B(t) \Bigr)+ \Bigl(\sup _{t\in[0,1]}a_{2}W_{\gamma}(t)-\inf _{t\in[0,1]}a_{2}W_{\gamma}(t) \Bigr) \\ &{}+ \Bigl(\sup_{t\in[0,1]}a_{3}B_{H}(t)-\inf _{t\in[0,1]}a_{3}B_{H}(t) \Bigr)+ \Bigl(\sup _{t\in[0,1]}a_{4}S_{\alpha}(t)-\inf _{t\in[0,1]}a_{4}S_{\alpha}(t) \Bigr) \\ ={}&\sup_{\mathrm{t}\in[0,1]^{4}} \bigl(a_{1}B(t_{1})+a_{2}W_{\gamma}(t_{2})+a_{3}B_{H}(t_{3})+a_{4}S_{\alpha}(t_{4}) \bigr) \\ &{}+\sup_{\mathrm{t}\in[0,1]^{4}} \bigl( \bigl(-a_{1}B(t_{1}) \bigr)+ \bigl(-a_{2}W_{\gamma}(t_{2}) \bigr)+ \bigl(-a_{3}B_{H}(t_{3}) \bigr)+ \bigl(-a_{4}S_{\alpha}(t_{4}) \bigr) \bigr) \\ \leq {}&\sup_{\mathrm{t}\in[0,1]^{4}} \bigl\vert a_{1}B(t_{1})+a_{2}W_{\gamma}(t_{2})+a_{3}B_{H}(t_{3})+a_{4}S_{\alpha}(t_{4}) \bigr\vert \\ &{}+\sup_{\mathrm{t}\in[0,1]^{4}} \bigl\vert a_{1}B(t_{1})+a_{2}W_{\gamma}(t_{2})+a_{3}B_{H}(t_{3})+a_{4}S_{\alpha}(t_{4}) \bigr\vert \\ ={}&2\sup_{\mathrm{t}\in[0,1]^{4}} \bigl\vert \mathbb{X}^{a_{1}, a_{2}, a_{3}, a_{4}}_{\gamma,H,\alpha}( \mathrm{t}) \bigr\vert . \end{aligned}$$

Hence

3.4 $$\begin{aligned} \mathbb{P} \Bigl(\sup_{\mathrm{t}\in[0,1]^{4}} \bigl\vert \mathbb{X}^{a_{1}, a_{2}, a_{3}, a_{4}}_{\gamma,H,\alpha}(\mathrm{t}) \bigr\vert \leq \varepsilon \Bigr)\leq {} &\mathbb{P} \Biggl(\sum _{i=1}^{4} \vert a_{i} \vert R_{i}\leq 2\varepsilon \Biggr) \\ \leq {}&\mathbb{P} \bigl( \vert a_{2} \vert R_{2} \leq 2\varepsilon \bigr). \end{aligned}$$

Combining (2.4) with (3.4), we obtain

$$\begin{aligned} \limsup_{\varepsilon\rightarrow 0^{+}}\varepsilon^{\beta}\log \mathbb{P} \Bigl(\sup_{\mathrm{t}\in [0,1]^{4}} \bigl\vert \mathbb{X}^{a_{1}, a_{2}, a_{3}, a_{4}}_{\gamma,H,\alpha}( \mathrm{t}) \bigr\vert \leq \varepsilon \Bigr)\leq -a_{2}^{2/(2\gamma+1)}k_{\gamma}. \end{aligned}$$

The proof of results in other conditions for Theorem 1.2 (1.1) follows from a similar approach given in the above argument, and we omit the details here. □

### Proof of (1.2) in Theorem 1.2

Fix $0<\varepsilon<1$, we note that

3.5 $$\begin{aligned} &\mathbb{P} \Bigl(\sup_{\mathrm{t}\in[0,1]^{4}} \bigl\vert \mathbb {Y}_{H,\gamma,\alpha}(\mathrm{t}) \bigr\vert \leq \varepsilon \Bigr) \\ &\quad \geq \mathbb{P} \Bigl(\sup_{t_{1}\in[0,1]} \bigl\vert B(t_{1}) \bigr\vert \cdot \sup_{t_{2}\in[0,1]} \bigl\vert W_{\gamma}(t_{2}) \bigr\vert \cdot \sup_{t_{3}\in[0,1]} \bigl\vert B_{H}(t_{3}) \bigr\vert \cdot \sup _{t_{4}\in[0,1]} \bigl\vert S_{\alpha}(t_{4}) \bigr\vert \leq \varepsilon ^{4} \Bigr) \\ &\quad \geq \mathbb{P} \Bigl( \Bigl\{ \sup_{t_{1}\in[0,1]} \bigl\vert B(t_{1}) \bigr\vert \leq \varepsilon \Bigr\} \cap \Bigl\{ \sup_{t_{2}\in[0,1]} \bigl\vert W_{\gamma}(t_{2}) \bigr\vert \leq \varepsilon \Bigr\} \\ &\qquad{} \cap \Bigl\{ \sup_{t_{3}\in[0,1]} \bigl\vert B_{H}(t_{3}) \bigr\vert \leq \varepsilon \Bigr\} \cap \Bigl\{ \sup_{t_{4}\in[0,1]} \bigl\vert S_{\alpha}(t_{4}) \bigr\vert \leq \varepsilon \Bigr\} \Bigr) \\ &\quad =\mathbb{P} \Bigl(\sup_{t_{1}\in[0,1]} \bigl\vert B(t_{1}) \bigr\vert \leq \varepsilon \Bigr)\cdot \mathbb{P} \Bigl(\sup_{t_{2}\in[0,1]} \bigl\vert W_{\gamma}(t_{2}) \bigr\vert \leq \varepsilon \Bigr) \\ &\qquad{}\times \mathbb{P} \Bigl(\sup_{t_{3}\in[0,1]} \bigl\vert B_{H}(t_{3}) \bigr\vert \leq \varepsilon \Bigr)\cdot \mathbb{P} \Bigl(\sup_{t_{4}\in[0,1]} \bigl\vert S_{\alpha}(t_{4}) \bigr\vert \leq \varepsilon \Bigr). \end{aligned}$$

Thus, combining (2.1), (2.3), (2.5), and (2.7)with (3.5), we obtain

$$\begin{aligned} \liminf_{\varepsilon\rightarrow 0^{+}}\varepsilon^{\beta}\log \mathbb{P} \Bigl(\sup_{\mathrm{t}\in[0,1]^{4}} \bigl\vert \mathbb{Y}_{\gamma,H,\alpha}( \mathrm{t}) \bigr\vert \leq \varepsilon \Bigr)\geq -\nu (\gamma, H, \alpha ), \end{aligned}$$

where $\nu (\gamma, H, \alpha )= (\operatorname{sgn}(2-\beta)+1 )\frac{\pi^{2}}{8} + (\operatorname{sgn}(2/(2\gamma+1)-\beta)+1 )k_{\gamma}+ (\operatorname{sgn}(1/H- \beta)+1 )C_{H} + (\operatorname{sgn}(\alpha-\beta)+1 )A_{\alpha}$. □

### Proof of (1.3) in Theorem 1.6

As a matter of convenience, we take $\vartheta (\gamma, H, \alpha, a_{1}, a_{2}, a_{3}, a_{4} )$ by abbreviated notation *ϑ*. Let $T_{n}=\theta^{n^{2}},\theta>1,n=1,\ldots $ . For any $\lambda<\vartheta^{1/\beta}$, using the scaling property and (1.1), we have

$$\begin{aligned} &\sum_{n=1}^{\infty}\mathbb{P} \Bigl(\sup _{\mathrm{t}\in [0,T_{n}]^{4}} \bigl\vert \mathbb{X}^{a_{1}, a_{2}, a_{3}, a_{4}}_{\gamma,H,\alpha}( \mathrm{t}) \bigr\vert \leq \lambda \bigl(T_{n}(\log\log T_{n})^{-1} \bigr)^{1/\beta} \Bigr) \\ &\quad =\sum_{n=1}^{\infty}\mathbb{P} \biggl( \sup_{\mathrm{t}\in[0,T_{n}]^{4}} \biggl\vert \frac{a_{1}B(t_{1}/T_{n})}{T_{n}^{-1/2}}+\frac{a_{2}W_{\gamma}(t_{2}/T_{n})}{T_{n}^{-(2\gamma+1)/2}}+ \frac{a_{3}B_{H}(t_{3}/T_{n})}{T_{n}^{-H}} \\ &\qquad{} +\frac{a_{4}S_{\alpha}(t_{4}/T_{n})}{T_{n}^{-1/\alpha}} \biggr\vert \leq \lambda \bigl(T_{n}(\log\log T_{n})^{-1} \bigr)^{1/\beta} \biggr) \\ &\quad \leq \sum_{n=1}^{\infty}\mathbb{P} \Bigl( \sup_{\mathrm{t}\in [0,1]^{4}} \bigl\vert \mathbb{X}^{a_{1}, a_{2}, a_{3}, a_{4}}_{\gamma,H,\alpha}( \mathrm{t}) \bigr\vert \leq \lambda (\log\log T_{n} )^{-1/\beta} \Bigr) \\ &\quad \leq \sum_{n=1}^{\infty}C(\lambda, \theta)\frac{1}{n^{2}} \\ &\quad < \infty. \end{aligned}$$

Then by the Borel–Cantelli lemma, we have

$$\begin{aligned} \liminf_{n\rightarrow\infty} \bigl(T_{n}^{-1}\log\log T_{n} \bigr)^{1/\beta}\sup_{\mathrm{t}\in[0,T_{n}]^{4}} \bigl\vert \mathbb{X}^{a_{1}, a_{2}, a_{3}, a_{4}}_{\gamma,H,\alpha}(\mathrm{t}) \bigr\vert \geq \lambda\quad \mbox{a.s.} \end{aligned}$$

For any $T_{n}\leq T\leq T_{n+1}$,

$$\begin{aligned} & \bigl(T^{-1}\log\log T \bigr)^{1/\beta}\sup _{\mathrm{t}\in[0,T]^{4}} \bigl\vert \mathbb{X}^{a_{1}, a_{2}, a_{3}, a_{4}}_{\gamma,H,\alpha}( \mathrm{t}) \bigr\vert \\ &\quad \geq \bigl(\theta^{-1/\beta}+o(1) \bigr) \bigl(T_{n}^{-1} \log \log T_{n} \bigr)^{1/\beta}\sup_{\mathrm{t}\in[0,T_{n}]^{4}} \bigl\vert \mathbb{X}^{a_{1}, a_{2}, a_{3}, a_{4}}_{\gamma,H,\alpha}(\mathrm{t}) \bigr\vert . \end{aligned}$$

Therefore

3.6 $$\begin{aligned} \liminf_{T\rightarrow\infty} \bigl(T^{-1}\log\log T \bigr)^{1/\beta}\sup_{\mathrm{t}\in[0,T]^{4}} \bigl\vert \mathbb{X}^{a_{1}, a_{2}, a_{3}, a_{4}}_{\gamma,H,\alpha}(\mathrm{t}) \bigr\vert \geq \theta^{1/\beta }\lambda \quad\mbox{a.s.} \end{aligned}$$

Thus the lower bound is proved by $\lambda\rightarrow\vartheta^{1/\beta}$ and $\theta\rightarrow1$ in (3.6).

Inspiration for the approach of the proof for the upper bound comes from Kuelbs [14] and Talagrand [15]. Taking $T_{k}=2^{k},k=1,\ldots $ . Let $\lambda>\vartheta^{1/\beta}$ and fix $\delta>0$. Then choose $j\geq 1$ independent of *k* such that $T_{k+j}\geq \delta^{-1}T_{k}$, and for every $k=1,\ldots $ ,

3.7 $$\begin{aligned} \bigl(T_{k+j}^{-1}\log\log T_{k+j} \bigr)^{1/\beta}< \delta \bigl(T_{k}^{-1}\log\log T_{k} \bigr)^{1/\beta}. \end{aligned}$$

Define the events

$$\begin{aligned} \mathrm{D}_{k}={}& \Bigl\{ \phi(T)\sup_{\mathrm{t}\in[0,T]^{4}} \bigl\vert \mathbb{X}^{a_{1}, a_{2}, a_{3}, a_{4}}_{\gamma,H,\alpha}(\mathrm{t}) \bigr\vert >\lambda \mbox{ for all } T\geq T_{k+j}, \phi(T_{k})\sup_{\mathrm{t}\in[0,T_{k}]^{4}} \bigl\vert \mathbb{X}^{a_{1}, a_{2}, a_{3}, a_{4}}_{\gamma,H,\alpha}(\mathrm{t}) \bigr\vert \leq \lambda \Bigr\} \hspace{-1pt}, \end{aligned}$$

where $\phi(T)= (T^{-1}\log\log T )^{1/\beta}$.

According to the definition of $\mathrm{D}_{k}$ and (3.7), we have

3.8 $$\begin{aligned} & \Bigl\{ \phi(T)\sup_{\mathrm{t}\in[T_{k},T]^{4}} \bigl\vert \mathbb{X}^{a_{1}, a_{2}, a_{3}, a_{4}}_{\gamma,H,\alpha}(\mathrm{t})- \mathbb{X}^{a_{1}, a_{2}, a_{3}, a_{4}}_{\gamma,H,\alpha}( \mathrm{T_{k}}) \bigr\vert >(1+\delta)\lambda \text{ for all } T \geq T_{k+j}, \\ &\quad \phi(T_{k})\sup_{\mathrm{t}\in[0,T_{k}]^{4}} \bigl\vert \mathbb{X}^{a_{1}, a_{2}, a_{3}, a_{4}}_{\gamma,H,\alpha}(\mathrm{t}) \bigr\vert \leq \lambda \Bigr\} \subset \mathrm{D}_{k}, \end{aligned}$$

where $\mathrm{T_{k}}= (T_{k},T_{k},T_{k},T_{k} )$.

Observe that

3.9 $$\begin{aligned} & \Bigl\{ \phi(T)\sup_{\mathrm{t}\in[T_{k},T]^{4}} \bigl\vert \mathbb{X}^{a_{1}, a_{2}, a_{3}, a_{4}}_{\gamma,H,\alpha}(\mathrm{t}) \bigr\vert >(1+2\delta) \lambda \text{ for all } T\geq T_{k+j}, \\ &\qquad{}{-}\phi(T) \bigl\vert \mathbb{X}^{a_{1}, a_{2}, a_{3}, a_{4}}_{\gamma,H,\alpha}( \mathrm{T_{k}}) \bigr\vert >-\delta\lambda \text{ for all } T\geq T_{k+j} \Bigr\} \\ &\quad \subset \Bigl\{ \phi(T)\sup_{\mathrm{t}\in[T_{k},T]^{4}} \bigl\vert \mathbb{X}^{a_{1}, a_{2}, a_{3}, a_{4}}_{\gamma,H,\alpha}(\mathrm{t})- \mathbb{X}^{a_{1}, a_{2}, a_{3}, a_{4}}_{\gamma,H,\alpha}( \mathrm{T_{k}}) \bigr\vert >(1+\delta)\lambda \text{ for all } T \geq T_{k+j} \Bigr\} \end{aligned}$$

and

3.10 $$\begin{aligned} & \Bigl\{ \phi(T_{k})\sup_{\mathrm{t}\in[0,T_{k}]^{4}} \bigl\vert \mathbb{X}^{a_{1}, a_{2}, a_{3}, a_{4}}_{\gamma,H,\alpha}(\mathrm{t}) \bigr\vert \leq \lambda \Bigr\} \\ &\quad \subset \bigl\{ -\phi(T) \bigl\vert \mathbb{X}^{a_{1}, a_{2}, a_{3}, a_{4}}_{\gamma,H,\alpha}( \mathrm{T_{k}}) \bigr\vert >-\delta\lambda \text{ for all } T\geq T_{k+j} \bigr\} . \end{aligned}$$

Therefore, using (3.8)–(3.10) and the scaling property, by the Gaussian correlation inequality [16] with any $0<\eta<1$, we obtain

$$\begin{aligned} \mathbb{P} (\mathrm{D}_{k} ) \geq {}& P_{k} \bigl( \bigl(1-\eta^{2} \bigr)^{1/2} \lambda \bigr)\\ &{}\times\mathbb{P} \Bigl(\phi(T)\sup_{\mathrm{t}\in[0,T-T_{k}]^{4}} \bigl\vert \mathbb{X}^{a_{1}, a_{2}, a_{3}, a_{4}}_{\gamma,H,\alpha}( \mathrm{t}) \bigr\vert > \eta(1+2\delta)\lambda \text{ for all } T\geq T_{k+j} \Bigr) \\ \geq {}& P_{k} \bigl( \bigl(1-\eta^{2} \bigr)^{1/2} \lambda \bigr)\\ &{}\times\mathbb{P} \Bigl(\phi(T)\sup _{\mathrm{t}\in[0,(1-\delta)T]^{4}} \bigl\vert \mathbb{X}^{a_{1}, a_{2}, a_{3}, a_{4}}_{\gamma,H,\alpha}( \mathrm{t}) \bigr\vert > \eta(1+2\delta)\lambda \text{ for all } T\geq T_{k+j} \Bigr) \\ \geq {}& P_{k} \bigl( \bigl(1-\eta^{2} \bigr)^{1/2} \lambda \bigr) \\ &{}\times \mathbb{P} \Bigl(\phi(T)\sup_{\mathrm{t}\in[0,T]^{4}} \bigl\vert \mathbb{X}^{a_{1}, a_{2}, a_{3}, a_{4}}_{\gamma,H,\alpha}(\mathrm{t}) \bigr\vert >(1- \delta)^{-1/\beta }\eta(1+2\delta)\lambda \text{ for all } T\geq T_{k+j} \Bigr), \end{aligned}$$

where $P_{k}((1-\eta^{2})^{1/2}\lambda)=\mathbb{P} (\phi(T_{k})\sup_{\mathrm{t}\in[0,T_{k}]^{4}} \vert \mathbb{X}^{a_{1}, a_{2}, a_{3}, a_{4}}_{\gamma,H,\alpha}(\mathrm{t}) \vert \leq (1-\eta ^{2})^{1/2}\lambda )$.

Hence, for every $N=1,\ldots $ , if only $T_{k}\geq N$, i.e., $k\geq \log N$,

$$\begin{aligned} \mathbb{P} (\mathrm{D}_{k} )\geq {}& P_{k} \bigl( \bigl(1- \eta^{2} \bigr)^{1/2}\lambda \bigr) \\ &{} \times\mathbb{P} \Bigl(\phi(T)\sup_{\mathrm{t}\in[0,T]^{4}} \bigl\vert \mathbb{X}^{a_{1}, a_{2}, a_{3}, a_{4}}_{\gamma,H,\alpha}(\mathrm{t}) \bigr\vert >(1- \delta)^{-1/\beta }\eta(1+2\delta)\lambda \text{ for all } T\geq N \Bigr). \end{aligned}$$

On the other hand, it is easy to see that the occurrence number of $\{{D_{k};k\geq 1}\}$ is no more than *j* by (3.7) and the definition of $D_{k}$, so there is

$$\sum_{k=1}^{\infty}\mathbb{P} ( \mathrm{D}_{k} )= \mathbb{E}\sum_{k=1}^{\infty}\mathrm{I}_{\mathrm{D}_{k}}\leq j. $$

Therefore

$$\begin{aligned} j\geq {}& \sum_{k\geq \log N}P_{k} \bigl( \bigl(1-\eta^{2} \bigr)^{1/2}\lambda \bigr) \\ &{}\times \mathbb{P} \Bigl(\phi(T)\sup_{\mathrm{t}\in[0,T]^{4}} \bigl\vert \mathbb{X}^{a_{1}, a_{2}, a_{3}, a_{4}}_{\gamma,H,\alpha}(\mathrm{t}) \bigr\vert >(1- \delta)^{-1/\beta }\eta(1+2\delta)\lambda \text{ for all } T\geq N \Bigr). \end{aligned}$$

Then, for any $(1-\eta^{2})^{1/2}\lambda>\vartheta^{1/\beta}$, by scaling

$$\begin{aligned} \sum_{k\geq \log N}P_{k} \bigl( \bigl(1- \eta^{2} \bigr)^{1/2}\lambda \bigr)=\sum _{k\geq \log N}\mathbb{P} \Bigl(\sup_{\mathrm{t}\in[0,T_{k}]^{4}} \bigl\vert \mathbb{X}^{a_{1}, a_{2}, a_{3}, a_{4}}_{\gamma,H,\alpha}(\mathrm{t}) \bigr\vert \leq \bigl(1-\eta^{2} \bigr)^{1/2}\lambda \phi^{-1}(T_{k}) \Bigr)=\infty. \end{aligned}$$

Accordingly, for every $N=1,\ldots $ , we have

$$\begin{aligned} \mathbb{P} \Bigl(\phi(T)\sup_{\mathrm{t}\in[0,T]^{4}} \bigl\vert \mathbb{X}^{a_{1}, a_{2}, a_{3}, a_{4}}_{\gamma,H,\alpha}(\mathrm{t}) \bigr\vert >(1- \delta)^{-1/\beta }\eta(1+2\delta)\lambda\text{ for all } T\geq N \Bigr)=0. \end{aligned}$$

Hence

3.11 $$\begin{aligned} \liminf_{T\rightarrow\infty} \bigl(T^{-1}\log\log T \bigr)^{1/\beta}\sup_{\mathrm{t}\in[0,T]^{4}} \bigl\vert \mathbb{X}^{a_{1}, a_{2}, a_{3}, a_{4}}_{\gamma,H,\alpha}(\mathrm{t}) \bigr\vert \leq (1- \delta )^{-1/\beta}\eta(1+2\delta)\lambda\quad \mbox{a.s.} \end{aligned}$$

The upper bound follows from (3.11) by $\delta\rightarrow 0$, $\eta\rightarrow1$ and $\lambda\rightarrow\vartheta^{1/\beta}$. □

## Concluding remarks

We end this paper with the following comment: it has been found that the small deviation estimate has close connection with various approximation quantities of compact sets and operators and has a variety of applications in studies of Hausdorff dimensions, rate of convergence in Strassen’s law of the iterated logarithm, and empirical processes (see, e.g., [17, 18]). Intuitively, we expect the results which were obtained in this paper can enrich the above relative fields. Moreover, there are scant papers related to the admixture additive processes and the admixture multiplicative processes. The behaviors of the admixture additive processes and the admixture multiplicative processes deserve to be investigated extensively.
